# Association of EEG Response to Hypertonic Saline and Neurologic Outcomes in Pediatric Acute Brain Injury

**DOI:** 10.1007/s12028-025-02422-x

**Published:** 2026-01-08

**Authors:** Emma L. Mazzio, Eva Catenaccio, Raymond Liu, Arastoo Vossough, Nicholas S. Abend, Alicia Alcamo, Jimmy W. Huh, Shih-Shan Lang, Robert A. Berg, Alexis A. Topjian, Craig Press, Matthew P. Kirschen

**Affiliations:** 1 Department of Anesthesiology and Critical Care Medicine, Children’s Hospital of Philadelphia and University of Pennsylvania, Philadelphia, PA, USA.; 2 Division of Pediatric Neurology, Departments of Neurology and Pediatrics, Children’s Hospital of Philadelphia and University of Pennsylvania, Philadelphia, PA, USA.; 3 Division of Neuroradiology, Children’s Hospital of Philadelphia and University of Pennsylvania, Philadelphia, PA, USA.; 4 Division of Neurosurgery, Children’s Hospital of Philadelphia and University of Pennsylvania, Philadelphia, PA, USA.

**Keywords:** Pediatrics, Brain injuries, Critical care, Quantitative EEG, Cerebral blood flow, Hypertonic saline, Prognosis

## Abstract

**Background:**

Electroencephalography (EEG) is a critical tool for neuromonitoring and neuroprognostication in children with acute brain injury. Quantitative EEG (qEEG), particularly the alpha-delta ratio (ADR), can detect worsening cerebral ischemia in adults, but it is unknown whether it can identify more subtle and transient changes in cerebral blood flow, such as those induced by hypertonic saline (HTS), in children with acute brain injury. We aimed to determine whether we could identify a cohort of patients with an ADR response to HTS and to evaluate the association between an ADR response and neurologic outcomes in critically ill children with acute brain injury.

**Methods:**

We conducted a retrospective cohort study of patients admitted to a pediatric intensive care unit with acute brain injury who received HTS during EEG monitoring from 2018 to 2023. The ADR was calculated before and after HTS administration. An ADR response was defined as a > 20% increase from baseline to within 30 min of receiving HTS in either hemisphere. The primary outcome was survival with favorable neurologic outcome, defined as a Functional Status Scale score change < 3 from prehospital baseline to discharge. Secondary outcome was survival to hospital discharge.

**Results:**

Among 87 patients (median age 10 years [interquartile range 3.6–14.5], 46% female), 28% (24 of 87) had an ADR response to HTS. ADR responders were older (12.9 vs. 8.0 years; *p* = 0.004) and more likely to have continuous, normal-voltage EEG backgrounds (67% vs. 40%; *p* = 0.006). Patients with an ADR response had four times increased odds of favorable outcome and survival (odds ratio [OR] 4.0 [95% confidence interval (CI) 1.3–12.7] and OR 3.9 [95% CI 1.0–10.7], respectively).

**Conclusions:**

An ADR increase > 20% following HTS was associated with increased odds of survival with favorable neurologic outcome and survival to hospital discharge in critically ill pediatric patients with acute brain injury. qEEG response to HTS may serve as a real-time, noninvasive biomarker of cerebral perfusion responsiveness.

## Introduction

Acute brain injury accounts for 15% of pediatric intensive care unit (PICU) admissions and is a leading cause of morbidity and mortality [1–4]. The acutely injured brain is highly vulnerable to secondary insults such as cerebral edema, elevated intracranial pressure, and impaired cerebral blood flow, all of which can exacerbate injury and worsen outcomes. Patients with acute brain injury frequently undergo continuous electroencephalographic (cEEG) monitoring to detect and guide management of subclinical seizures [5–8]. Additionally, EEG background features are biomarkers of acute brain injury severity. More normal background features such as continuous activity, the presence of faster frequencies, and the presence of variability are associated with favorable neurologic outcomes [9–16]. Further, tracking the trajectory of a patient’s EEG background over the course of an illness can provide insight into the severity of the disease process and response to therapies and inform prognosis [17, 18].

Quantitative EEG (qEEG) allows for objective assessment of EEG background activity by transforming raw EEG signals into analytic trends that quantify changes in amplitude and frequency content over time. Frequency-domain metrics, including alpha (8–13 Hz), beta (13–30 Hz), theta (4–8 Hz), and delta (1–4 Hz) power, are among the most commonly used in critical care and have been associated with both cerebral injury severity and neurologic outcomes in pediatric populations [19–22]. The alpha-delta ratio (ADR), a commonly used qEEG metric in adult neurocritical care, reflects the relative balance of faster (alpha) and slower (delta) frequency components. Cerebral blood flow is one of many factors that modulates ADR; reductions in cerebral blood flow lead to a loss of faster frequencies and a predominance of slower waveforms, yielding a decreased ADR [23–26]. In critically ill adults, ADR is sensitive to clinically apparent ischemic events such as delayed cerebral ischemia following subarachnoid hemorrhage [21, 27–38].

Hypertonic saline (HTS) is commonly administered to patients to manage intracranial hypertension, potentially improving cerebral blood flow through osmotic shifts, reduction in blood viscosity, and plasma volume expansion [39–41]. It is unclear whether changes in ADR are demonstrable after more subtle and transient increases in cerebral blood flow, such as those induced by HTS. Furthermore, it is unknown whether transient increases in ADR have prognostic significance in children with acute brain injury. Thus, in a cohort of children with acute brain injury, we aimed to determine whether administration of clinically indicated 3% HTS was associated with an increase in ADR and whether there was an association between ADR response to HTS and favorable neurologic outcome. We hypothesized that patients with an ADR response to HTS would have higher rates of survival with favorable neurologic outcome compared to patients without an ADR response. Lastly, we evaluated the relationship between a sustained versus a transient ADR response to HTS and outcome, hypothesizing that patients with a sustained ADR response would have an increased likelihood of survival with a favorable neurologic outcome.

## Methods

This was a single-center retrospective observational cohort study of patients admitted to the PICU with an acute brain injury who received at least one dose of HTS as part of clinical care while undergoing cEEG monitoring from January 1, 2018, to July 31, 2023. Exclusion criteria included the following: (1) younger than three months of age; (2) excessive artifact, as determined by an epileptologist, rendering it unsuitable for quantitative analysis; and (3) insufficient EEG data surrounding the analyzed HTS dose. The study was approved by the Children’s Hospital of Philadelphia Institutional Review Board, and a waiver of consent was obtained for this retrospective review of clinically collected data.

We obtained clinical characteristics, including pre-ICU admission baseline and hospital discharge Functional Status Scale (FSS) scores from a local Virtual Pediatric Systems database, entered on admission by trained critical care nurses [42]. The FSS is a validated scale of functional status, with scores ranging from 6 to 30; 6 represents normal functional status, and higher scores demonstrate worse functional status. We abstracted the timing and dose of HTS, along with pre- and post-administration sodium and carbon dioxide (CO_2_) values, from the electronic medical record. Pre-HTS values were defined as the sodium and CO_2_ measurements obtained within 3 h prior to HTS administration, selecting the value closest to the time of administration. Post-HTS values were the most proximal measurements within 3 h after administration. CO_2_ levels were preferentially obtained from arterial or venous blood gases. If unavailable, we used hourly recorded end-tidal CO_2_ values. For each patient, only the first dose of 3% HTS administered during cEEG monitoring was included in the analysis. At our institution, 3% HTS was administered at a standard dose of 2–5 mL/kg, and no other concentrations were used. Patients may have received a dose of HTS prior to cEEG monitoring or had insufficient EEG data surrounding a prior dose for analysis (e.g., EEG disconnected to obtain neuroimaging). The neuroimaging study obtained closest to, and within 24 h of, the HTS dose was independently reviewed by a pediatric neuroradiologist (AV). The severity of acute brain injury was categorized as normal (no acute injury), moderate (injury not meeting severe criteria), or severe (presence of midline shift or herniation).

cEEG was performed as part of clinical care using a standard 10–20 electrode montage for all patients (Natus, version 9.3.1 2013). A board-certified pediatric electroencephalographer (EC), blinded to the qEEG metrics, reviewed all raw EEG tracings to ensure the segments surrounding HTS doses were free of artifact that could impact qEEG analysis and that no seizures were present. EEG backgrounds were classified into three categories based on standard critical care EEG definitions: (1) continuous with normal voltage, (2) continuous or nearly continuous with low or normal voltage, and (3) discontinuous, burst suppression, or suppressed patterns [9, 10, 13, 17, 18, 43, 44]. Additionally, EEG reports were reviewed for the presence of reactivity (yes/no/not available) within 24 h of HTS administration. Reports without mention of reactivity or where reactivity could not be assessed were coded as “not available.”

Raw EEG signals were processed in Persyst (Version 15, Persyst Development Corporation, Prescott, AZ) using artifact reduction and fast Fourier transformation analysis to compute power spectral density estimates. Alpha (8–13 Hz) and delta (1–4 Hz) power were extracted using a running average over 8-s epochs. The ADR for each hemisphere was calculated as the quotient of alpha power divided by delta power. We abstracted the ADR for a total of 40 min, extending from 5 min prior to the HTS dose to 30 min following the 5-min infusion duration. Baseline ADR was the average ADR over the 5-min period before HTS administration. Post-HTS ADR was calculated as the average ADR during each of the following time intervals: 0–10, 11–20, and 21–30 min following HTS administration. ADRs were calculated separately for each hemisphere for all time points. An ADR response to HTS was defined as an increase of greater than 20% from baseline in either hemisphere during at least one of the three post-HTS time intervals. Among patients with an ADR response, the response was categorized as sustained or transient. A sustained response was defined as an ADR increase of > 20% that persisted for at least two consecutive time intervals (totaling at least 20 min) or from onset through the end of the 30-min post-HTS monitoring interval; all other responses were classified as transient responders. The 20% threshold was extrapolated from prior studies using ADR to detect delayed cerebral ischemia in adult subarachnoid hemorrhage, hemispheric differences in stroke, and cerebral blood flow changes during neonatal aortic arch reconstruction [28, 29, 32, 34–36, 38].

The primary outcome was survival with a favorable neurologic outcome, defined as survival with an FSS score change of < 3 from pre-ICU admission to hospital discharge [45]. The secondary outcome was survival to hospital discharge.

We report descriptive statistics as medians and interquartile ranges (IQRs) for continuous variables and as frequencies with percentages for categorical variables. We used the χ^2^ test or Fisher’s exact test to examine associations between categorical variables and outcome, and we used the Wilcoxon rank-sum test to compare continuous variables between exposure and outcome groups. All statistical tests were two-sided, and *p* < 0.05 was considered statistically significant. Univariable logistic regression was used to assess the association between ADR response and outcome. Because normal EEG frequency composition varies by age, ADR responses were evaluated in age-based subgroups (< 3 years and ≥ 3 years), consistent with prior work demonstrating developmental increases in alpha power after 3 years of age [19]. As a secondary analysis we stratified patients based on EEG background category to assess if the association between ADR response and outcome differed by EEG background. We conducted two sensitivity analyses to exclude patients who (1) received a dose of HTS prior to cEEG initiation and (2) had acute hypoxic-ischemic brain injury.

## Results

Among 151 patients who met inclusion criteria, 64 were excluded due to age less than 3 months (*n* = 4), excessive EEG artifact (*n* = 7), or insufficient EEG data (*n* = 53). Thus, 87 patients were analyzed. The median age was 10.4 years (IQR 3.6–14.5), and 46% were female. An ADR response to HTS occurred in 28% of patients (24 of 87). Compared to ADR nonresponders, ADR responders were older (12.9 [IQR 10.5–15.0] vs. 8.0 [IQR 2.2–12.2] years; *p* = 0.004), but the two groups did not differ in other demographics or illness severity metrics (Table 1). ADR responders were more likely to have a continuous and normal-voltage EEG background compared to ADR nonresponders (67% vs. 40%; *p* = 0.01). Median dose of HTS was similar between responders and nonresponders (5.0 [IQR 4.3–5.0] vs. 4.6 [IQR 3.0–5.0] mL/kg; *p* = 0.28). Changes in serum sodium and CO_2_ levels pre- and post-HTS dose were similar between ADR responder groups (Table 1).

Survival with a favorable neurologic outcome occurred in 17% of patients (15 of 87), and survival to hospital discharge occurred in 66% of patients (58 of 87) (Tables 2, 3). More ADR responders survived with a favorable neurologic outcome than ADR nonresponders (33% vs. 11%; *p* = 0.03). Having an ADR response was associated with four times increased odds of favorable neurologic outcome (odds ratio [OR] 4.0, 95% confidence interval [CI] 1.3–12.7). Survival to hospital discharge was higher among ADR responders compared to nonresponders (83% vs. 60%; *p* = 0.05), corresponding to 3.9 times greater odds of survival (OR 3.9, 95% CI 1.0–10.7).

Among ADR responders, 25% of patients (6 of 24) demonstrated a response onset within the 0–10-min interval, 66% (16 of 24) demonstrated a response onset during the 11–20-min interval, and 8% (2 of 24) demonstrated a response onset during the 21–30-min interval. The ADR response was sustained in 75% of patients (18 of 24) and transient in 25% of patients (6 of 24). Among patients who survived with a favorable neurologic outcome, a greater proportion had a sustained versus transient ADR response, although the difference was not statistically significant (39% vs. 17%; OR 3.2 [95% CI 0.3–33.6]).

When stratified by EEG background category, the association between ADR response and favorable neurologic outcome remained directionally consistent but was not statistically significant across strata (Table 4). In patients with continuous and normal-voltage EEG, survival with favorable neurologic outcome occurred in 44% of ADR responders compared to 28% of ADR nonresponders (OR 2.0 [95% CI 0.5–7.7]). Among the 46 patients with continuous low-voltage/nearly continuous and discontinuous EEG backgrounds, only one patient survived with favorable neurologic outcome. EEG reactivity was present in 28 of 64 patients (44%) and was not associated with ADR response (*p* = 0.83).

Forty-two percent (37 of 87) of patients received at least one dose of HTS prior to cEEG initiation. When evaluating the 50 patients whose first dose of HTS was captured on cEEG, ADR responders had higher rates of survival with favorable neurologic outcomes than ADR nonresponders (38% vs. 8%; OR 7.08 [95% CI 1.39–35.99]). When evaluating patients without hypoxic-ischemic brain injury (*n* = 63), there was no difference in rates of favorable neurologic outcome (44% vs. 17%; OR 2.5 [95% CI 0.7–8.4]) or survival to hospital discharge (90% vs. 71%; OR 3.8 [95% CI 0.76–18.9]) among ADR responders and ADR nonresponders.

## Discussion

In a cohort of critically ill children with acute brain injury, patients with a > 20% increase in ADR within 30 min of HTS administration had four times the odds of survival with a favorable neurologic outcome compared to ADR nonresponders. The association between an ADR response and favorable neurologic outcome was strongest among patients with more normal EEG backgrounds (continuous and normal voltage), highlighting a subgroup with likely less severe injury in whom a response to HTS may more readily translate into clinical benefit. An increase in faster EEG frequencies (alpha) or a reduction in slower frequencies (delta) following HTS administration likely reflects improved cerebral blood flow in salvageable brain tissue [21, 26–28]. In our cohort, only one child under 3 years of age demonstrated an ADR response, perhaps reflecting both the severity of injury in this subgroup and developmental differences in EEG frequency composition. Younger children have lower alpha power, and measures incorporating theta frequencies may better capture cerebral changes in this age group. In addition, open cranial sutures may reduce the likelihood of pressure-limited cerebral blood flow during acute injury, making an ADR response to HTS less apparent.^19^ Although we hypothesized that sustained ADR response would be associated with favorable outcome, this was not observed and may reflect the clinical importance of the presence of an ADR response rather than the duration of the ADR response. Taken together, these findings suggest that an ADR response to HTS may potentially serve as a noninvasive biomarker of neurovascular responsiveness in acute brain injury, identifying patients with the potential to benefit from strategies that improve cerebral blood flow.

ADR can be calculated automatically in real-time and displayed continuously at the bedside, allowing clinicians to evaluate EEG changes in response to interventions aimed at improving cerebral physiology. The qEEG response, or lack of response, to an intervention may provide information about the physiologic state of the brain that can guide subsequent neuroprotective therapies. For example, the presence of an ADR response to HTS may suggest further therapies aimed at improving cerebral blood flow could be beneficial. On the other hand, the absence of an ADR response may identify children with (1) less severe brain injury and preserved cerebral perfusion, (2) more severe intracranial hypertension in whom a single dose of HTS is insufficient and more aggressive ICP management is needed, or (3) irreversible brain injury unlikely to respond to hyperosmolar therapy in whom alternative ICP management strategies should be considered. Although qEEG and serial ADR monitoring are commonly used in adult neurocritical care and have demonstrated high sensitivity (83–100%) and specificity (74–84%) for detecting delayed cerebral ischemia in aneurysmal subarachnoid hemorrhage [29, 34–37], it is an emerging tool for neuromonitoring in pediatric populations [20, 22, 27, 30, 31]. For example, in neonates undergoing congenital heart surgery, the development of an interhemispheric ADR difference greater than 25% has been associated with subsequent neurologic injury [27, 28]. Our findings build on this prior work in adults and pediatrics by demonstrating the value of a detectable, real-time qEEG response to a physiologic intervention.

Identifying patients with an ADR response following HTS may help understand the cerebral physiological and likelihood of clinical benefit from additional hyperosmolar therapy. There is mixed evidence and large practice variation in the use of HTS in patients with primarily cytotoxic cerebral edema from etiologies such as hypoxic-ischemic brain injury from cardiac arrest, infectious encephalitis, and ischemic strokes. The use of HTS in hypoxic-ischemic brain injury decreases brain edema in animal models but has failed to show improvement in clinical studies [46, 47]. However, recent work suggests that in hypoxic-ischemic brain injury, HTS may reduce perivascular edema and thereby improve brain tissue oxygenation, which may be beneficial for a subset of patients with less malignant EEG patterns [48, 49]. In our cohort, only one of the 24 patients with hypoxic-ischemic brain injury achieved a favorable outcome, and this patient was an ADR responder. In this context, an EEG-based marker like ADR responsiveness may offer a physiologic tool to differentiate patients likely to benefit from HTS from those who may require alternative neuroprotective management strategies.

EEG background following pediatric acute brain injury is a strong predictor of outcome [5, 9–11, 50]. In our study, nearly all children who survived with favorable neurologic outcome exhibited continuous and normal-voltage EEG backgrounds. Although not statistically significant, the presence of an ADR response appeared to further stratify patients with continuous EEG backgrounds, with double the percentage of patients experiencing favorable outcomes who had both a continuous EEG background and an ADR response to HTS compared to patients with a continuous EEG background who did not have an ADR response to HTS. Continuous, more normal EEG backgrounds are more likely to demonstrate reactivity to external stimuli, such as a bedside examination, which could result in an increase in ADR. However, when reactivity was assessed within 24 h of HTS administration, there was no association between the presence of reactivity and ADR response, suggesting that the ADR change is independent of EEG reactivity. Among patients with continuous, normal-voltage EEGs who did not demonstrate an ADR response, it is possible that some had relatively mild injuries with preserved cerebral perfusion such that HTS did not lead to an increase in cerebral blood flow to elicit EEG changes. Among 46 patients with continuous low-voltage/nearly continuous and discontinuous EEG backgrounds, only one patient survived with a favorable neurologic outcome, limiting the ability to evaluate the association between ADR responsiveness and outcome within this group. That patient was an ADR responder. Taken together, these findings suggest that ADR response should not be used in isolation to predict neurologic outcome and that larger studies are needed to better define the relationship between ADR response and EEG background features.

Our study has several limitations. The retrospective, single-center design and modest sample size limited our ability to perform multivariable regression analyses. Our cohort was composed of patients who received HTS doses while on EEG, often using a patient’s second or third dose for analyses, potentially underestimating the observed association, as the efficacy of HTS may wane with repeated doses. We could not account for how a prior response to HTS influenced subsequent administrations. In addition, the indication for HTS administration was not consistently documented, limiting our ability to distinguish between doses given for intracranial hypertension and those administered to achieve sodium targets. EEG background categorization was based on a 5-min epoch immediately preceding HTS administration. Although this short interval was chosen to capture the acute cerebral physiology at the time clinicians made the decision to give HTS, it risks potential misclassification of a patient’s true baseline ADR, which may have been more evident if a longer baseline interval was used. We did not control for baseline neurologic conditions, antiepileptic medication use, or preexisting encephalopathy, all of which could influence EEG background frequencies. Because the abundance of alpha frequencies increases with age, our results may underestimate responsiveness in children aged less than 3 years [19]. We did not evaluate other concurrent clinical interventions performed during episodes of suspected intracranial hypertension, including sedative boluses or application of painful stimuli, that may influence EEG frequency power and contribute to observed ADR changes. Highfrequency intracranial and cerebral perfusion pressures at the time of analyzed HTS administration were only available for three patients, precluding evaluation of ADR responses with decreases in intracranial pressure or increases in cerebral perfusion pressure. Finally, the 20% ADR threshold was extrapolated from prior studies of cerebral ischemia in adults and children, but prospective studies are needed to determine optimal cut points in children [28, 29, 32, 34–36, 38].

## Conclusions

A greater than 20% increase in ADR within 30 min of HTS administration was associated with increased survival with favorable neurologic outcome in critically ill children with acute brain injury. Quantitative EEG response to physiologic-targeted interventions is a promising noninvasive tool to aid in both prognostication and therapeutic decision-making in pediatric acute brain injury.

## Figures and Tables

**Table 1:** Patient demographic and clinical characteristics by ADR response to HTS

	All patients (*N* = 87)	ADR responder (*n* = 24)	ADR nonresponder (*n* = 63)	*p* value
Age, median (IQR), years	10.4 (3.6–14.5)	12.9 (10.6–15.0)	8.0 (2.2–12.2)	0.004
< 3 years	21 (24%)	1 (4%)	20 (32%)	0.009
≥ 3 years	66 (76%)	23 (96%)	43 (68%)	
Female	40 (46%)	15 (63%)	25 (40%)	0.09
Pre-ICU FSS, median (IQR)	6 (6–6)	6 (6–6)	6 (6–6)	0.82
PRISM3 score, median (IQR)	11 (5–17)	11 (5–15)	11 (6–18)	0.55
Previously healthy	57 (66%)	18 (75%)	39 (62%)	0.37
*Etiology of acute brain injury*				
Anoxic	24 (28%)	3 (13%)	21 (33%)	0.08
Trauma	24 (28%)	9 (38%)	15 (24%)	
ICH nontraumatic	15 (17%)	5 (38%)	10 (16%)	
Neuroinflammatory/infectious	12 (14%)	3 (13%)	9 (14%)	
Metabolic	4 (6%)	2 (8%)	2 (3%)	
Ischemic stroke	4 (6%)	0 (0%)	4 (6%)	
Status epilepticus	2 (2%)	2 (8%)	0 (0%)	
Other	2 (2%)	0 (0%)	2 (3%)	
*EEG background category*				
Cont., normal voltage	41 (47%)	16 (67%)	25 (40%)	0.006
Cont./nearly cont. + /− low voltage	23 (26%)	7 (29%)	32 (51%)	
Discont./suppressed/burst suppression	23 (26%)	1 (4%)	6 (10%)	
EEG reactivity (*n* = 64)	28 (32%)	7 (29%)	21 (33%)	0.83
*Neuroimaging severity (n = 81)*				
Normal	9 (10%)	2 (8%)	7 (11%)	0.35
Mild to moderate	46 (53%)	17 (71%)	30 (46%)	
Severe	25 (29%)	5 (21%)	20 (32%)	
Dose of HTS (mL/kg)	4.8 (3.1–5.0)	5.0 (4.3–5.0)	4.6 (3.0–5.0)	0.28
Pre-HTS sodium (*n* = 85)	139 (135–145)	142 (139–145)	138 (134–145)	0.06
Δ Sodium (*n* = 84)	2 (1–4)	2 (1–4)	2 (1–4)	0.75
Pre-HTS CO_2_ (*n* = 85)	36 (33–39)	35 (31–38)	36 (33–40)	0.14
Δ CO_2_ (*n* = 85)	0 (−2 −2)	1 (−3–2)	0 (−2–2)	0.81

*ADR* alpha-delta ratio, *CO*_*2*_ carbon dioxide level from blood gas, *Cont*. continuous, *Discont*. discontinuous, *EEG* electroencephalography, *FSS* functional status score, *HTS* hypertonic saline, *ICH* intracranial hemorrhage, *ICU* intensive care unit, *IQR* interquartile range, *PRISM3* pediatric risk of mortality score 3, Δ sodium, pre-/post-HTS change, Δ CO_2_, pre-/post-HTS change in CO_2_ from blood gas or EtCO_2_

**Table 2:** Association between ADR response to HTS and favorable neurologic outcome

	All patients (*N* = 87)	ADR responder (*n* = 24)	ADR nonresponder (*n* = 63)	*p* value	Odds ratio (95% CI)
Favorable neurologic outcome	15 (17%)	8 (33%)	7 (11%)	0.03	4.0 (1.3–12.7)
Survival	58 (66%)	20 (83%)	38 (60%)	0.05	3.9 (1.0–10.7)

Association between ADR response and favorable neurologic outcome and survival to hospital discharge

*ADR* alpha-delta ratio, *CI* confidence interval, *HTS* hypertonic saline

**Table 3:** Patient demographic and clinical characteristics by primary outcome

	Favorable neurologic outcome (*n* = 15)	Nonfavorable neurologic outcome (*n* = 72)	*p* value
Age, median (IQR), years	10.4 (6.9–13.9)	10.4 (2.8–14.5)	0.32
< 3 years	1 (7%)	20 (28%)	0.10
≥ 3 years	14 (93%)	52 (72%)	
Female	9 (60%)	31 (43%)	0.36
Male	6 (40%)	41 (57%)	
Baseline FSS	6 (6–6)	6 (6–6)	0.29
PRISM3 score	5 (5–15)	13 (8–18)	0.001
Previously healthy	8 (53%)	49 (68%)	0.37
Etiology of acute brain injury			0.01
Hypoxic ischemic	1 (7%)	23 (32%)	
Trauma	2 (13%)	22 (30%)	
ICH nontraumatic	4 (27%)	11 (15%)	
Neuroinflammatory/infectious	4 (27%)	8 (11%)	
Metabolic	3 (20%)	1 (1%)	
Ischemic stroke	0 (0%)	4 (6%)	
Status epilepticus	1 (7%)	1 (1%)	
Other	0 (0%)	2 (3%)	
EEG background category			< 0.001
Cont. normal voltage	14 (93%)	27 (38%)	
Cont./nearly cont. + /− low voltage	1 (7%)	22 (31%)	
Discont./suppressed/burst suppression	0 (0%)	23 (32%)	
Neuroimaging severity			0.16
Normal	3 (20%)	6 (8%)	
Mild to moderate	10 (67%)	37 (51%)	
Severe	2 (13%)	23 (32%)	

*Cont*. continuous, *Discont*. discontinuous, *EEG* electroencephalography, *FSS* functional status score, *ICH* intracranial hemorrhage, *IQR* interquartile range, *PRISM3* pediatric risk of mortality score 3

**Table 4:** Association between ADR response to HTS and outcome, stratified by EEG background

	All (*n* = 41)	ADR responder (*n* = 16)	ADR nonresponder (*n* = 25)	*p* value	OR (95% CI)
*Continuous, normal-voltage EEG background*					
Favorable neurologic outcome	14 (34%)	7 (44%)	7 (28%)	0.48	2.0 (0.5–7.7)
Survival	35 (85%)	15 (93%)	20 (80%)	0.36	3.3 (0.4–94.8)
	All (*n* = 23)	ADR responder (*n* = 7)	ADR nonresponder (*n* = 16)	*p* value	OR (95% CI)
*Continuous or nearly continuous +/− low voltage*					
Favorable neurologic outcome	1 (4%)	1 (14%)	0 (0%)	0.30	-
Survival	15 (65%)	5 (71%)	10 (62%)	1.00	1.4 (0.21–13.98)
	All (*n* = 23)	ADR responder (*n* = 1)	ADR nonresponder (*n* = 22)	*p* value	OR (95% CI)
*Discontinuous, burst suppressed, suppressed*					
Favorable neurologic outcome	0	0	0	–	–
Survival	8 (34%)	0	8	1.00	–

Comparison of favorable neurologic outcome and survival to hospital discharge among patients with and without an ADR response to HTS, stratified by EEG background category

*ADR* alpha-delta ratio, *CI* confidence interval, *EEG* electroencephalography, *HTS* hypertonic saline, *OR* odds ratio
